# Clinical Presentation and Molecular Characteristics of Kabuki Syndrome With Congenital Hyperinsulinism: A Retrospective Study

**DOI:** 10.7759/cureus.101532

**Published:** 2026-01-14

**Authors:** Mélanie Gaudillière, Thibaud Armand, Valérie Senée, Carine Villanueva, Marc Nicolino, Kevin Perge

**Affiliations:** 1 Department of Pediatric Endocrinology, Diabetology and Metabolism, Hôpital Femme-Mère-Enfant, Hospices Civils de Lyon, Lyon, FRA; 2 Department of Genetics, Centre de Biologie et de Pathologie Est, Hospices Civils de Lyon, Paris, FRA; 3 Department of Genetics, Institut Cochin, Institut National De La Santé Et De La Recherche Médicale, Université Paris Cité, Paris, FRA

**Keywords:** diazoxide, hyperinsulinism, hypoglycaemia, kabuki syndrome, kmt2d

## Abstract

Introduction: Kabuki syndrome (KS) is a rare disease predisposing to congenital hyperinsulinism (CHI). The incidence of CHI in KS may be higher than considered in practice, and appropriate management of hypoglycemia would reduce long-term neurologic morbidity in these patients. Thus, the aims of our study were to estimate the occurrence rate of CHI-related hypoglycaemia in KS, to describe its evolution, and to identify potential genotype-phenotype correlations.

Methods: We conducted a single-centre retrospective study in patients with KS from 1999 to 2024.

Results: Among the 18 patients identified with KS included in the study, six (33%) presented neonatal hypoglycaemia, of whom five (28%) demonstrated CHI. Of these five, three had persistent CHI. Only the three patients with this condition required anti-hypoglycaemic drugs. They all responded well to diazoxide monotherapy, and one then needed the addition of octreotide. Genetically, all patients had heterozygous variants of *the KMT2D *gene except one, who had a heterozygous microdeletion of the same gene. Among these 17 variants, the majority were truncating variants (10/17, 59%) (three nonsense variants, six frameshift variants, and one splicing variant). Seven variants were missense variants (7/17, 41%). Among the six patients with hypoglycaemia, all had nonsense or frameshift variants in the C-terminal part of the KMT2D protein.

Conclusions: This study illustrates the importance of systematic screening for hypoglycaemia in patients with early diagnosis of KS. Conversely, newborns or infants with CHI should be considered and possibly tested for this etiological diagnosis.

## Introduction

Described for the first time in 1981 by Kuroki et al. [1] and Niikawa et al. [2], Kabuki syndrome (KS) is an uncommon and variable congenital condition, occurring in roughly one out of 32,000 births in Japan and about one out of 86,000 in other regions of the world [3,4]. Genetically, KS demonstrates an autosomal dominant pattern of inheritance and two genes have been identified: *KMT2D* discovered in 2010 [5], located on chromosome 12q13, which variants are found in 70-75% of cases [6], and *KDM6A*, first described in 2012 [7], located on Xp11.23, which variants are found in 1-9% of cases [6].

Clinically, this syndrome presents five principal traits: a facial dysmorphy (prominent ears, arched eyebrows with the lateral one third dispersed, depressed nasal tip, and neversion of the lower lateral eyelid), various skeletal anomalies such as brachydactyly and scoliosis, distinctive dermatoglyphic patterns including persistent fingertip pads and an increased number of loop fingerprints, mild to moderate cognitive impairment, and postnatal growth delay despite normal weight and length at birth [3,4,8]. Other commonly reported clinical features include neonatal hypotonia, recurrent otitis media, feeding problems, cleft palate, oligodontia, congenital heart defect, gastrointestinal and renal anomalies, immune deficiency, and congenital hypothyroidism [4].

Hypoglycaemia is an uncommon endocrine manifestation [9,10], and when it occurs, it is often linked to growth hormone deficiency, adrenal or adrenocorticotropic hormone (ACTH) insufficiency, or to congenital hyperinsulinism (CHI) [11,12]. CHI can be transient or permanent and is often described as a rare manifestation, characterized by a good response to diazoxide. Because recent studies have shown that the incidence of CHI would be more important than considered in practice [11] and because an appropriate management of hypoglycaemias would reduce long-term neurologic morbidity in these patients [12], we conducted a retrospective study in patients affected with KS in our department of paediatric endocrinology in order to: (i) estimate the occurrence rate of CHI-related hypoglycaemia in KS, (ii) to describe its evolution, and (iii) to identify potential genotype-phenotype correlations.

## Materials and methods

This was a retrospective study of all children with genetically proven KS followed between 1999 and 2024 in the Department of Paediatric Endocrinology, Hôpital Femme Mère Enfant under Hospices Civils de Lyon, Lyon, France. The study protocol received approval from the Scientific and Ethical Committee of Hospices Civils de Lyon and was carried out in accordance with the principles of the Declaration of Helsinki. In line with current French regulations, parents were informed about the study.

Molecular genetics analyses

Genomic DNA was extracted from ethylenediaminetetraacetic acid (EDTA)-treated peripheral blood samples using the Maxwell® 16 LEV Blood DNA Kit (Promega, Madison, Wisconsin, United States). The exons and exon-intron junctions of the *KMT2D* gene were first examined using Sanger sequencing or targeted next-generation sequencing (MiSeq; Illumina, Inc., San Diego, California, United States).

Data collection

Information on genotypes (variant type, molecular nature, and predicted protein impact), demographic characteristics (sex, ethnicity, and age at last evaluation), and clinical and biochemical phenotypes was collected for each case. Children were considered cured of CHI from the time they no longer required hyperglycaemic medication and had normal carbohydrate intakes for their age.

Definitions

Hypoglycaemia was defined as a plasma glucose value < 50 mg/dL, and CHI diagnosis was established as an excessive and inappropriate level of insulin during episodes of hypoglycaemia (insulin level >1 mUI/L). If CHI lasted more than six months, it was considered persistent.

Statistical analysis

Phenotypic and genotypic characteristics were examined using descriptive statistical methods. Continuous quantitative variables were summarized by their median and interquartile range (IQR), while categorical variables were described using frequency distributions.

## Results

Patient characteristics

**Figure 1:**
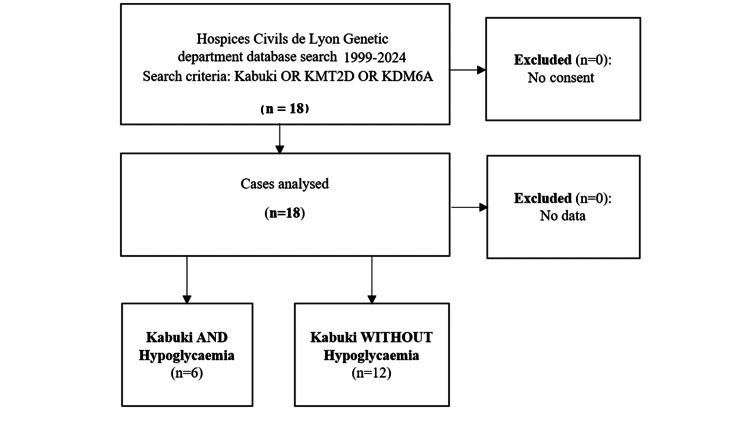
Flowchart of study participants

A total of 18 patients with KS followed up between 1999 and 2024 were included in our study (Figure 1). Table 1 and Table 2 summarize the clinical characteristics of these patients. Among the 18 patients (11 boys (61%) and seven girls (39%)) with KS studied, 33% (n=6) presented with neonatal hypoglycaemia, of which 28% (n=5) demonstrated CHI. In all the cases, hypoglycaemia was diagnosed on day 1, with the diagnosis of KS evoked in all patients, particularly in the presence of the facial phenotype. There were no children with intrauterine growth retardation and no mothers with gestational diabetes. Three of the KS patients (17%) had persistent CHI; only these three patients required anti-hypoglycaemic drugs, and they all responded well to diazoxide monotherapy, and one then needed the addition of octreotide. In the overall cohort, the median birth weight was 2910 g (range, 2687-3385 g) and the median age at delivery was 39 weeks of gestation (range, 38 weeks-39 weeks+5 days). The children were mostly of Caucasian origin (72%). No clinical differences were obvious at birth between those who develop hypoglycaemia and those who do not.

**Table 1:** Clinical characteristics of KS patients and comparison of characteristics according to the presence of hypoglycaemia Gestational age was determined either from the last menstrual period with a 15-day correction to approximate the date of conception, or from first-trimester ultrasound dating when available. IUGR: intrauterine growth restriction; CHI: congenital hyperinsulinism; KS: Kabuki syndrome

Parameters	Total cohort (n=18), n (%)	Patients with hypoglycaemia (n=6), n (%)	Patients without hypoglycaemia (n=12), n (%)
Sex male	11 (61%)	3 (50%)	8 (67%)
Sex female	7 (39%)	3 (50%)	4 (33%)
IUGR	0 (0%)	0 (0%)	0 (0%)
Median birth weight (grams), median (IQR)	2910 (2687-3385)	2910 (2595-3710)	3005 (2687-3305)
Age at delivery (weeks+day of gestation)	39 (38-39+5)	38 (36+6-40)	39 (38-39+5)
Caucasian ethnicity	13 (72%)	5 (83%)	8 (67%)
CHI confirmed	5 (28%)	5 (83%)	0 (0%)

**Table 2:** Clinical and laboratory characteristics of patients included in the study. CEF: continuous enteral feeding; D: day; DM: maltose dextrin; F: female; Hypo: hypoglycaemia; I: insulinemia (mUI/L); IEF: intermittent enteral feeding; IV: intravenous infusion; M: male; NA: not available; NG tube: nasogastric tube; PG: plasma glucose (mg/dL)

Case ID	Age at last investigation (years)	Sex	Ethnicity	Age at delivery (weeks of gestation)	Birth weight (grams)	Presentation of hypoglycaemia/Level of PG (mg/dL)	Insulinemia (mUI/L)	Treatment of hypoglycaemia	Evolution	Variants
1	18	M	Maghreb	39	3200	0	-	0	0	Gene *KMT2D* Exon 31 De novo c.7216G>A p.Asp2406Asn
2	14	M	Caucasian	40	3260	0	-	0	0	Gene *KMT2D* Exon 39 De novo g.13040_13041del p.Gln4347Argfs* 23
3	8	F	Caucasian	39	2770	Hypo D1 PG: 32mg/dL	NA	CEF +IV (7,5mg/kg/min) then IEF +DM by NG TUBE stop D12	0	Gene *KMT2D* Exon 3 De novo c.269_272del p.Asp90Glyfs*39
4	16	F	Caucasian	38	2790	0	-	0	0	Gene *KMT2D* Exon 51 De novo c.16273G>A p.Glu5425Lys
5	11	M	Caucasian	37+6	3420	Hypo D1 PG :21mg/dL	4	CEF+IV (8mg/kg/min) then IEF +DM by NG TUBE stop D10	0	Gene *KMT2D* Exon 48 De novo c.14946dupG p.Lys4983Glufs*24
6	10	M	African/ Caucasian	38	2920	Hypo D1 PG :35mg/dL	6	CEF +IV (12mg/kg/min) +Diazoxide 8mg/kg/d from D14, then IEF DM by NG TUBE, then IEF by gastrostomy from 6 months	Stop Diazoxide at 6 years old	Gene *KMT2D* Exon 39 De novo c.11515dup p.Gln3839Profs*173
7	11	F	Caucasian	39+2	3680	0	-	0	0	Gene *KMT2D* Exon 48 De novo c.15640C>G p.Arg 5214Gly
8	4	F	Caucasian	41	4000	Hypo D1 PG: 28mg/dL	5	CEF +IV (9.5mg/kg/min) then IEF +DM by NG TUBE stop D21	Stop NG TUBE at 6 months old	Gene *KMT2D* Exon 47 De novo c.14568G>A p.Trp 4856*
9	19	M	Caucasian	40	3520	0	-	0	0	Gene *KMT2D* Exon 39 De novo c.11791C>T p.Leu3931Phe
10	11	M	Caucasian	35	2420	0	-	0	0	Gene *KMT2D* Exon 48 De novo c.15461G>A p.Arg5154Gln
11	19	F	Maghreb	38	2700	0	-	0	0	Gene *KMT2D* Exon 53 NS De novo g.16501C>T p.Arg5501*
12	22	F	Caucasian	39	3350	0	-	0	0	Gene *KMT2D *Exon 28 De novo c.5993A>G p.Tyr1998Cys
13	7 months	F	Caucasian	37+5	2900	Hypo D1 PG :34mg/dL	12	CEF +IV(10mg/kg/min) + Diazoxide 7mg/kg/d from D21, then IEF +DM by NG TUBE, then IEF by gastrostomy from 5 months old	IEF with 11mg/kg/d + Diazoxide 9mg/kg/d	Gene *KMT2D* Exon 39 De novo c.11944C>T p.Arg3982*
14	21 months	M	Maghreb/ Turkish	40	3160	0	-	0	0	Gene *KMT2D* Micodélétion 15q24 1.6Mb
15	17	M	Caucasian	36	2420	Hypo D1 PG :23mg/dL	8	CEF +IV (11mg/kg/min)+ glucagon +diazoxide 10mg/kg/d from D14+ octreotide by pump from 3 months old, then IEF by gastrostomy from 6 months old	Stop diazoxide at 4 years Stop octreotide at 10 years	Gene *KMT2D* Exon 31 De novo c.6987_6988insT p.Pro2330Serfs*47
16	14	M	Turkish	39+2	2850	0	-	0	0	Gene *KMT2D *Intron 46 De novo c.14516-1G>C p.Gly4840Glufs*30
17	12	M	Caucasian	37	2600	0	-	0	0	Gene *KMT2D* Exon 31 De novo c.6751del p.Ser2251Argfs*13
18	15	M	Caucasian	38+2	2625	0	-	0	0	Gene *KMT2D* Exon 31 De novo c.7670C>T p.Pro2557Leu

KS cases with CHI

Case 3

Patient 3 was a girl born to healthy, unrelated parents. She was born at term and eutrophic (39 AW, 2770 g). She presented hypoglycaemia on the first day of life without a determined aetiology (plasma glucose at 32 mg/dl, insulin level not available). She was able to achieve normoglycaemia through continuous glucose intake via nasogastric tube and intravenous infusion (intakes around 7,5mg/kg/min), then by discontinuous intakes that were enriched with maltose dextrin administered with nasogastric tube. She recovered from hypoglycaemia without any pharmacological treatment on day 12. Genetic samples revealed a heterozygous de novo variant in *the KMT2D *gene (c.269_272del) leading to a truncated protein (p.Asp90Glyfs*39).

Case 5

Patient 5 was a boy born to healthy, unrelated parents. He was born at term and eutrophic (37+6 AW, 3420 g). He presented hypoglycaemia on the first day of life (insulin level at 4 mUI/L for plasma glucose at 21 mg/dl). He was able to achieve normoglycaemia through continuous glucose intake via nasogastric tube and intravenous infusion (intakes around 8 mg/kg/minute), then by discontinuous intakes that were enriched with maltose dextrin administered with a nasogastric tube. He recovered from hypoglycaemia without any pharmacological treatment on day 10. Genetic samples revealed a heterozygous de novo variant* KMT2D* gene (c.14946dupG), leading to a truncated protein (p.Lys4983Glufs*24).

Case 6

Patient 6 was a boy born to healthy, unrelated parents. He was born at term and eutrophic (38 AW, 2920 g). He presented hypoglycaemia on the first day of life (insulin level at 6 mUI/L for plasma glucose at 35 mg/dl). He was able to achieve normoglycaemia through continuous glucose intake via nasogastric tube and intravenous infusion (intakes around 12 mg/kg/minute), then by diazoxide therapy from day 14 at 8 mg/kg/day. Then, he had discontinuous intakes that were enriched with maltose dextrin administered with a nasogastric tube, then with a gastrostomy. He recovered from hypoglycaemia at the age of six months. Genetic samples revealed a heterozygous de novo variant *KMT2D* gene (c.11515dup), leading to a truncated protein (p.Gln3839Profs*173).

Case 8

Patient 8 was a girl born to healthy, unrelated parents. She was born at term and eutrophic (41 AW, 4000 g). She presented hypoglycaemia on the first day of life (insulin level at 5 mUI/L for plasma glucose at 28 mg/dl). She was able to achieve normoglycaemia through continuous glucose intake via nasogastric tube and intravenous infusion (intakes around 9,5 mg/kg/minute), then by discontinuous intakes that were enriched with maltose dextrin administered with nasogastric tube. She recovered from hypoglycaemia without any pharmacological treatment on day 21. Genetic samples revealed a heterozygous de novo variant *KMT2D* gene (c.14568G>A), leading to a truncated protein (p.Trp4856*).

Case 13

Patient 13 was a girl born to healthy, unrelated parents. She was born at term and eutrophic (37+5 AW, 2900 g). She presented hypoglycaemia on the first day of life (insulin level at 12 mUI/L for plasma glucose at 34 mg/dl). She was able to achieve normoglycaemia through continuous glucose intake via a nasogastric tube and intravenous infusion (intakes around 10 mg/kg/minute), then by diazoxide therapy from day 21 at 7 mg/kg/day. Then, she had discontinuous intakes that were enriched with maltose dextrin administered by nasogastric tube, then by gastrostomy. She was still being treated with diazoxide at a dose of 9 mg/kg/day and glucose intakes were around 11 mg/kg/minute. Genetic samples revealed a heterozygous de novo variant *KMT2D* gene (c.11944C>7), leading to a truncated protein (p.Arg3982*).

Case 15

Patient 15 was a boy born to healthy, unrelated parents. He was born at term and eutrophic (36 AW, 2420 g). He presented hypoglycaemia on the first day of life (insulin level at 8 mUI/L for plasma glucose at 23 mg/dl). He was able to achieve normoglycaemia through continuous glucose intake via nasogastric tube and intravenous infusion (intakes around 11 mg/kg/minute), then by diazoxide therapy from day 14 at 10 mg/kg/day, and finally by using octreotide infused subcutaneously by pump from the age of three months. Then, he had discontinuous intakes that were enriched with maltose dextrin administered by nasogastric tube, then by gastrostomy. He recovered from hypoglycaemia at 10 years old. Diazoxide was stopped at four years of age, and octreotide at 10 years old. Genetic samples revealed a de novo variant *KMT2D* gene (c.6987_6988insT; p.Pro2330Serfs*47).

Genotype/phenotype correlations

KS is inherited in an autosomal dominant manner. For all the cases, each specific mutation was not found in any of the parents’ DNA and therefore considered to be a de novo mutation. They had all heterozygous variants of *the KMT2D* gene except one, who had a heterozygous microdeletion of the same gene (Table 4). Among these 17 variants, the majority were truncating variants (10/17, 59%) (three nonsense variants (3/17, 18%), six frameshift variants (6/17, 35%), and one splicing variant (1/17, 6%). Seven variants (41%) were missense variants. Among the six patients with hypoglycaemia, all had nonsense or frameshift variants in the C-terminal part of the KMT2D protein. In other words, 55% (6/11) of patients with heterozygous truncating variants or deletion in *KMT2D* presented hypoglycaemia compared to 0% (0/7) in patients with missense variants. 

**Table 3:** Presentation, therapeutic management, and evolution of hypoglycaemia in patients with a KMT2D gene mutation. CEF: continuous enteral feeding; D: day; DM: maltose dextrin; Hypo: hypoglycaemia; I: insulinemia (mUI/L); IEF: Intermittent enteral feeding; IV: intravenous infusion; NA: not available; NG tube: nasogastric tube; PG: plasma glucose (mg/dL)

Case ID	Presentation of hypoglycaemia/Level of PG (mg/dL)	Insulinemia (mUI/L)	Treatment of hypoglycaemia	Evolution
3	Hypo D1 PG: 32mg/dL	NA	CEF +IV (7,5mg/kg/min) then IEF +DM by NG TUBE	Stop at D12
5	Hypo D1 PG: 21mg/dL	4	CEF+IV (8mg/kg/min) then IEF +DM by NG TUBE	Stop at D10
6	Hypo D1 PG: 35mg/dL	6	CEF +IV (12mg/kg/min) + Diazoxide 8mg/kg/d from D14, then IEF+ DM by NG TUBE, then IEF by gastrostomy from 6 months old	Stop Diazoxide at 6 years old
8	Hypo D1 PG: 28mg/dL	5	CEF +IV (9.5mg/kg/min) then IEF +DM by NG TUBE	Stop at D21
13	Hypo D1 D1 :34mg/dL	12	CEF +IV (10mg/kg/min) + Diazoxide 7mg/kg/d from D21, then IEF +DM by NG TUBE, then IEF by gastrostomy from 5 months old	IEF with 11mg/kg/d + Diazoxide 9mg/kg/d
15	Hypo D1 D1: 23mg/dL	8	CEF +IV (11mg/kg/min)+ +Diazoxide 10mg/kg/d from D14+ octreotide by pump from 3 months old, then IEF by gastrostomy from 6 months old	Stop Diazoxide at 4 years old Stop octreotide at 10 years old

**Table 4:** Genetic characteristics of patients with KS (KMT2D - NM_003482.3) FS: frameshift; I: intronic splicing variants; MS: missense; NS: nonsense; KS: Kabuki syndrome

Case	Exon	Nucleotide change	Amino acid change	Type
1	31	c.7216G>A	p.Asp2406Asn	MS
2	39	c.13040_13041del	p.Gln4347Argfs*23	FS
3	3	c.269_272del	p.Asp90Glyfs*39	FS
4	51	c.16273G>A	p.Glu5425Lys	MS
5	48	c.14946dup	p.Lys4983Glufs*24	FS
6	39	c.11515dup	p.Gln3839Profs*173	FS
7	48	c.15640C>G	p.Arg5214Gly	MS
8	47	c.14568G>A	p.Trp4856*	NS
9	39	c.11791C>T	p.Leu3931Phe	MS
10	48	c.15461G>A	p.Arg5154Gln	MS
11	53	c.16501C>T	p.Arg5501*	NS
12	28	c.5993A>G	p.Tyr1998Cys	MS
13	39	c.11944C>T	p.Arg3982*	NS
14	All	Microdeletion 1.6Mb		Del
15	31	c.6987_6988insT	p.Pro2330Serfs*47	FS
16	-	c.14516-1G>C	p.Gly4840Glufs*30	I
17	31	c.6751del	p.Ser2251Argfs*13	FS
18	31	c.7670C>T	p.Pro2557Leu	MS

## Discussion

Neurologic outcomes can be compromised in neonates with hyperinsulinemic hypoglycaemia. Thus, early recognition and appropriate treatment are vital to minimize neurocognitive impairment in particular [13]. In KS, hypoglycaemia is often described as a rare manifestation, occurring in around 6.5-10% of cases according to different studies [14-16]. In our cohort, interestingly, the rate of hypoglycaemia was between three and four times higher, while there was no history of gestational diabetes observed in mothers or IUGR, both situations being also at risk for neonatal hypoglycaemia. CHI is not reported as a major feature of KS in the literature, even if KS is described as the second most common syndromic form of CHI [10,11]. In our study, CHI was found in a quarter of KS cases and was associated with hypoglycaemias except in one of the cases where hypoglycaemia remained without aetiology. Moreover, we had three patients who presented with persistent CHI, representing half of the patients with CHI and 17% of the global KS cohort, whereas this persistent form is described as occurring rarely in KS patients [14].

Concerning the genetic variants found in* the KMT2D* gene for the hypoglycaemic patients, just over half were nonsense and frameshift variants. This result was in favour of the causal role of the altered structure of the KMT2D protein as suggested in a previous study [17]. Indeed, the* KMT2D* gene has 54 coding exons and encodes a large protein, which is a histone- lysine-N-methyltransferase of 5537 amino acids that belongs to the Trithorax group of proteins [18]. KMT2D functions as a transcriptional activator by modifying histones to stimulate the expression of its target genes, and itseems to participate in regulating cytoskeleton- and adhesion-associated processes that can influence cellular growth and survival [19]. However, the mechanism through which *KMT2D* pathogenic variants would result in CHI remains to be determined by further research. [20].

In terms of genotype-phenotype correlations, our study found that only the patients with heterozygous truncating variants in *KMT2D* may present hypoglycaemia compared to patients with missense variants. Moreover, most of the variants responsible for CHI were localized on the C-terminus part of the protein, which must play an important role in the functionality of the protein, as had already been noted in the study of Makrythanansis et al. [16]. On the other hand, there is no clear genotype-phenotype correlation with regard to the transient versus persistent character of CHI. In our cohort, all patients demonstrated a pathogenic variant in *the KMT2D* gene. This finding aligns with published data, as most individuals with KS carry variants of this type, whereas only 2-8% of cases are reported to harbour pathogenic variants in the *KDM6A* gene [6]. It is important to note that all our patients with CHI had pathogenic variants in *the KMT2D* gene, whereas CHI is more commonly associated with *KDM6A* variants [4,6,21,22]. The mechanism by which haploinsufficiency of the *KDM6A* gene causes CHI in KS is not fully understood, but it appears to involve disruption of epigenetic changes during pancreatic differentiation [23]. Pathogenic variants in the *KDM6A* gene could cause pancreatic beta cell dysfunction, via possible mechanisms including altered KATP channel function or altered adenosine triphosphate (ATP)/adenosine diphosphate (ADP) ratio.

In general, reports of hypoglycaemia in children indicate that, without appropriate management, affected patients may develop severe neurological complications such as seizures, developmental delay, or even cerebral palsy. Diazoxide remains the first-line pharmacological treatment for CHI and is effective in controlling hypoglycaemia, particularly in patients with the persistent form of the disease [17]. In our study, only the three patients with this condition required anti-hypoglycaemic drugs. They all responded well to diazoxide monotherapy, and one then needed the addition of octreotide, as similarly described in other previous publications [10,24]. 

The main limitations of the study are its retrospective nature and the absence of a systematic investigation of hypoglycemia and etiological assessment.

## Conclusions

Our study highlights that hypoglycaemia, most often related to CHI, is a more common feature of KS. Importantly, persistent forms of CHI, although typically considered rare in KS, accounted for half of CHI cases in our series. These findings underline the need for systematic glucose monitoring in all neonates with early clinical suspicion or confirmed diagnosis of KS, regardless of birth weight or maternal history. From a molecular perspective, our data point toward a possible genotype-phenotype correlation, with hypoglycaemia occurring exclusively in patients carrying truncating variants of *KMT2D*, mostly located in the C-terminal region of the protein. These findings reinforce the essential functional role of this domain in pancreatic physiology and may contribute to improving risk stratification in KS. Nonetheless, larger multicentre studies are needed to confirm this association and to clarify the mechanisms by which KMT2D dysfunction leads to impaired insulin secretion.

Overall, our work emphasizes the importance of raising awareness among neonatologists, endocrinologists, and clinical geneticists that Kabuki syndrome represents a significant cause of syndromic CHI. Conversely, infants presenting with unexplained hyperinsulinaemic hypoglycaemia should prompt consideration of KS and appropriate genetic testing. Improving early detection of this association is key to optimizing neurodevelopmental outcomes and guiding genetic counselling for affected families.

## References

[1] Kuroki Y, Suzuki Y, Chyo H, Hata A, Matsui I (1981). A new malformation syndrome of long palpebral fissures, large ears, depressed nasal tip, and skeletal anomalies associated with postnatal dwarfism and mental retardation. J Pediatr.

[2] Niikawa N, Matsuura N, Fukushima Y, Ohsawa T, Kajii T (1981). Kabuki make-up syndrome: a syndrome of mental retardation, unusual facies, large and protruding ears, and postnatal growth deficiency. J Pediatr.

[3] Ng SB, Bigham AW, Buckingham KJ (2010). Exome sequencing identifies MLL2 mutations as a cause of Kabuki syndrome. Nat Genet.

[4] Banka S, Veeramachaneni R, Reardon W (2012). How genetically heterogeneous is Kabuki syndrome?: MLL2 testing in 116 patients, review and analyses of mutation and phenotypic spectrum. Eur J Hum Genet.

[5] Li Y, Bögershausen N, Alanay Y (2011). A mutation screen in patients with Kabuki syndrome. Hum Genet.

[6] Bögershausen N, Gatinois V, Riehmer V (2016). Mutation update for Kabuki syndrome genes KMT2D and KDM6A and further delineation of X-linked Kabuki syndrome subtype 2. Hum Mutat.

[7] Lederer D, Grisart B, Digilio MC (2012). Deletion of KDM6A, a histone demethylase interacting with MLL2, in three patients with Kabuki syndrome. Am J Hum Genet.

[8] Adam MP, Banka S, Bjornsson HT (2019). Kabuki syndrome: international consensus diagnostic criteria. J Med Genet.

[9] Bereket A, Turan S, Alper G, Comu S, Alpay H, Akalin F (2001). Two patients with Kabuki syndrome presenting with endocrine problems. J Pediatr Endocrinol Metab.

[10] Subbarayan A, Hussain K (2014). Hypoglycemia in Kabuki syndrome. Am J Med Genet A.

[11] De Leon DD, Stanley CA (2017). Congenital hypoglycemia disorders: new aspects of etiology, diagnosis, treatment and outcomes: highlights of the proceedings of the congenital hypoglycemia disorders symposium, Philadelphia, April 2016. Pediatr Diabetes.

[12] Maiorana A, Dionisi-Vici C (2017). Hyperinsulinemic hypoglycemia: clinical, molecular and therapeutical novelties. J Inherit Metab Dis.

[13] Vajravelu ME, De León DD (2018). Genetic characteristics of patients with congenital hyperinsulinism. Curr Opin Pediatr.

[14] Geneviève D, Amiel J, Viot G (2004). Atypical findings in Kabuki syndrome: report of 8 patients in a series of 20 and review of the literature. Am J Med Genet A.

[15] Shangguan H, Su C, Ouyang Q, Cao B, Wang J, Gong C, Chen R (2019). Kabuki syndrome: novel pathogenic variants, new phenotypes and review of literature. Orphanet J Rare Dis.

[16] Makrythanasis P, van Bon BW, Steehouwer M (2013). MLL2 mutation detection in 86 patients with Kabuki syndrome: a genotype-phenotype study. Clin Genet.

[17] Gohda Y, Oka S, Matsunaga T, Watanabe S, Yoshiura K, Kondoh T, Matsumoto T (2015). Neonatal case of novel KMT2D mutation in Kabuki syndrome with severe hypoglycemia. Pediatr Int.

[18] FitzGerald KT, Diaz MO (1999). MLL2: a new mammalian member of the trx/MLL family of genes. Genomics.

[19] Issaeva I, Zonis Y, Rozovskaia T (2007). Knockdown of ALR (MLL2) reveals ALR target genes and leads to alterations in cell adhesion and growth. Mol Cell Biol.

[20] Banka S, Lederer D, Benoit V (2015). Novel KDM6A (UTX) mutations and a clinical and molecular review of the X-linked Kabuki syndrome (KS2). Clin Genet.

[21] Gole H, Chuk R, Coman D (2016). Persistent hyperinsulinism in Kabuki syndrome 2: case report and literature review. Clin Pract.

[22] Lindgren AM, Hoyos T, Talkowski ME (2013). Haploinsufficiency of KDM6A is associated with severe psychomotor retardation, global growth restriction, seizures and cleft palate. Hum Genet.

[23] Li C, Ackermann AM, Boodhansingh KE (2017). Functional and metabolomic consequences of K(ATP) channel inactivation in human islets. Diabetes.

[24] Yap KL, Johnson AE, Fischer D (2019). Congenital hyperinsulinism as the presenting feature of Kabuki syndrome: clinical and molecular characterization of 9 affected individuals. Genet Med.

